# DNA damage response and evasion from immunosurveillance in CLL: new options for NK cell-based immunotherapies

**DOI:** 10.3389/fgene.2015.00011

**Published:** 2015-02-04

**Authors:** Olga M. Shatnyeva, Hinrich P. Hansen, Katrin S. Reiners, Maike Sauer, Maulik Vyas, Elke Pogge von Strandmann

**Affiliations:** Innate Immunity Group, Clinic 1 for Internal Medicine, University of Cologne, Cologne, Germany

**Keywords:** chronic lymphocytic leukemia, DNA damage response, natural killer cell, immunotherapy, immunoligands, immunoconstructs

## Abstract

Chronic lymphocytic leukemia (CLL) is the most prominent B cell malignancy among adults in the Western world and characterized by a clonal expansion of B cells. The patients suffer from severe immune defects resulting in increased susceptibility to infections and failure to generate an antitumor immune response. Defects in both, DNA damage response (DDR) pathway and crosstalk with the tissue microenvironment have been reported to play a crucial role for the survival of CLL cells, therapy resistance and impaired immune response. To this end, major advances over the past years have highlighted several T cell immune evasion mechanisms in CLL. Here, we discuss the consequences of an impaired DDR pathway for detection and elimination of CLL cells by natural killer (NK) cells. NK cells are considered to be a major component of the immunosurveillance in leukemia but NK cell activity is impaired in CLL. Restoration of NK cell activity using immunoligands and immunoconstructs in combination with the conventional chemotherapy may provide a future perspective for CLL treatment.

## INTRODUCTION

Chronic lymphocytic leukemia (CLL) is an indolent lymphoproliferative disorder characterized by the progressive accumulation of monoclonal CD5^+^ B cells in the peripheral blood, bone marrow and secondary lymphoid tissues (Ghia and Hallek, 2014; Yair et al., 2014). Another typical feature of CLL is extraordinary high frequency of chromosomal aberrations often associated with the DNA damage response (DDR) pathway and dysregulation of the cell cycle (Landau et al., 2013). Recent data suggest that defects of the DDR pathway and interaction with bystander cells of the microenvironment are pivotal factors for CLL progression. Prolonged DNA damage and defective repair results in the release of DNA-HMGB1 complexes from necrotic cells and the induction of inflammatory response that can be hijacked by CLL cells (Jia et al., 2014).

The standard therapy of CLL which includes combined chemotherapy and/or immunotherapy is highly efficient for the depletion of CLL cells from the peripheral blood but not from lymphoid tissue and bone marrow. Moreover, this might result in the selection of resistant clones (Landau et al., 2013). Haploidentical stem cell transplantation (HSCT) is an alternative treatment but restricted to a limited group of patients due to lack of suitable donors and may cause fatal side effects. New successful therapeutic strategies include modulation of the microenvironment and activation of the patient immune system to combat cancer cells (Burger and Gribben, 2014; Yair et al., 2014).

Direct targeting of tumor-associated antigens (TAA) on malignant cells by monoclonal antibodies (mAb) is regarded as a promising approach (Simpson and Caballero, 2014). Thus, antibodies against CD20 (rituximab, Obinutuzumab), CD19 (GBR 401), CD23 (lumiliximab), or CD52 (alemtuzumab) are currently evaluated (Robak, 2013). More recently, immunotherapies with genetically engineered chimeric T-cell receptors (CARs), which detect TAA, were developed (Burger and Gribben, 2014; Yair et al., 2014). So far, T cells with specificity for the common B cell antigens CD19, CD20, and CD23 were generated (Riches and Gribben, 2013). Initial clinical trials revealed feasibility, and increasingly also impressive antitumor effects.

There is emerging evidence that natural killer (NK) cells also play a pivotal role in the immunosurveillance of CLL (Reiners et al., 2013; Huergo-Zapico et al., 2014). Understanding of the molecular mechanisms of evasion from NK cell-mediated immune responses and recovery of their function will help to develop novel treatment strategies. In this review we focus on the role of DDR defects and the immune microenvironment in the evasion from NK cell responses in CLL, as far as on restoration of NK cell function using immunoligands and immunoconstructs.

## THE ROLE OF DDR DEFECTS IN CLL DEVELOPMENT: IMPACT ON ESCAPE FROM NK CELL IMMUNE RESPONSE

Recent studies have identified 20 candidate CLL driver genes associated with core signaling pathways, including DNA repair, cell cycle control, Notch signaling, inflammatory pathways, Wnt signaling, RNA splicing, and RNA processing (Landau et al., 2013), among which mutations associated with DNA repair and cell cycle regulation are most recurrent.

The physiological function of the DDR pathway is to detect DNA damage, to signal its presence and to mediate DNA repair. The proximal DDR constitutes of two major kinase branches, the ATM/Chk2 and the ATR/Chk1 pathways. Activation of ATR, which phosphorylates its effector kinase Chk1, is induced in response to single-strand breaks and bulky DNA lesions. The ATM kinase, signaling through its effector Chk2, is activated primarily in response to DNA double-strand breaks (DSBs), such as those induced by alkylating agents, topoisomerase inhibitors, or ionizing radiation. Chk1 and Chk2 have a protective function providing time to the cell to repair genotoxic lesions. Both kinase pathways result in activation of cell cycle-arresting target genes, DNA repair and apoptosis via p53 activation (Reinhardt and Yaffe, 2009).

Recent sequencing studies identified recurrent somatic gene mutations in CLL patients for proteins involved in DNA damage signaling and DNA repair, including mutations in TP53, ATM, CHEK1, CHEK2, POT1, BRCA1, and CHD2 (Puente et al., 2011; Quesada et al., 2011).

The ATM-Chk2-p53 signaling axis plays an important role in regulation of apoptotic response to DNA damage in CLL, as mutations in ATM and TP53 are enriched in patients with secondary resistance to DNA-damaging chemotherapy (Bartkova et al., 2005; Landau et al., 2013).

ATM gene is frequently inactivated in CLL and is associated with defective apoptosis in response to chemotherapeutic agents (Austen et al., 2007). ATM mutant cells exhibit impaired DNA DSB repair. Poly (ADP-ribose) polymerase (PARP) plays a pivotal role in a direct repair of DSBs and involved in main DNA repair mechanisms: homologous recombination and non-homologous end-joining (Weston et al., 2010). ATM dysfunction is associated with significantly higher PARP activity in CLL patients, which might mediate genomic instability and progression of the disease. *In vivo* studies using xenograft model of an ATM mutant cell line demonstrated significantly reduced tumor load and an increased survival of animals after treatment with the PARP inhibitor Olaparib (Weston et al., 2010). Clinical studies with Olaparib demonstrated sufficient efficacy in patients with ATM deficient, relapsed and refractory CLL (ISRCTN34386131 DOI 10.1186/ISRCTN34386131). Deletions of the short arm of chromosome 17 (del(17p)) where TP53 is located are found in 5–8% of chemotherapy-naïve patients (Dohner et al., 2000). Mutations of TP53 are found in 4–37% of patients with CLL, and have been associated with very poor prognosis (ultra-high risk) in a number of studies (Zenz et al., 2010). Among cases with confirmed del(17p), the majority show mutations in the remaining TP53 allele (>80%). Higher genomic complexity and clinical diversity of CLL are associated with T53 mutations. Impaired DDR promotes a “mutator phenotype,” which allows the acquisition of additional genetic lesions driving transformation in CLL (Seiffert et al., 2012).

Mutational inactivation of the DDR is an established hallmark of CLL and associated with high genomic instability (Zenz et al., 2010; Landau et al., 2013).

ATM appears to be a major regulator of the p53 response. They communicate the genotoxic lesion to the apoptotic machinery but they are frequently inactivated in CLL and are associated with poor response to conventional chemotherapy (ten Hacken and Burger, 2014).

The B cell receptor (BCR) pathway inhibitors in CLL have shown high efficacy in the cases with poor chromosomal aberrations such as Del (17p) or p53 mutation, known to acquire resistance to standard chemotherapy. Downstream targets of the BCR such as SYK, Bruton’s tyrosine kinase (BTK), or PI3K isoform p110 delta have a promising anti-neoplastic activity in patients with CLL. Responses are typically manifested by rapid regression of enlarged lymph nodes and splenomegaly that is accompanied by transient lymphocytosis (Burger and Gribben, 2014; Yair et al., 2014).

Clinical trials with Idelalisib, PI3K delta isoform inhibitor, have a dramatic and durable response in CLL patients with a markers of poor prognosis, such as mutations in p53, ATM and NOTCH1. Monotherapy with Idelalisib and combination with other therapeutical agents such as Rituximab and Ofatumumab results show good activity in CLL regardless of high-risk prognostic markers (Khan et al., 2014).

Moreover, the DDR is able to alert the immune system toward the stressed cell, mainly through the recruitment of NK cells, which are able to identify and eliminate dangerous cells without prior antigen-mediated stimulation (Raulet, 2006; Bryceson and Ljunggren, 2008). NK cells do not only distinguish between “self” and “non-self,” but specifically seek for pathological changes in endogenous cells. One important danger signal is the inducible expression of ligands for cytotoxic NK cell receptors [NKG2D (NK group 2, member D) and NCRs (natural cytotoxicity receptors)] to alarm the innate immune system in response to DNA damage (Gasser et al., 2005; Gasser and Raulet, 2006a,b,c; Gasser, 2007; Soriani et al., 2009; Fine et al., 2010; Norman et al., 2011). Ligands for these NK cell receptors are not expressed on normal cells but are found on cells undergoing cellular stress that causes DNA damage including chemotherapeutics or ionizing radiation (Raulet, 2006).

**FIGURE 1:**
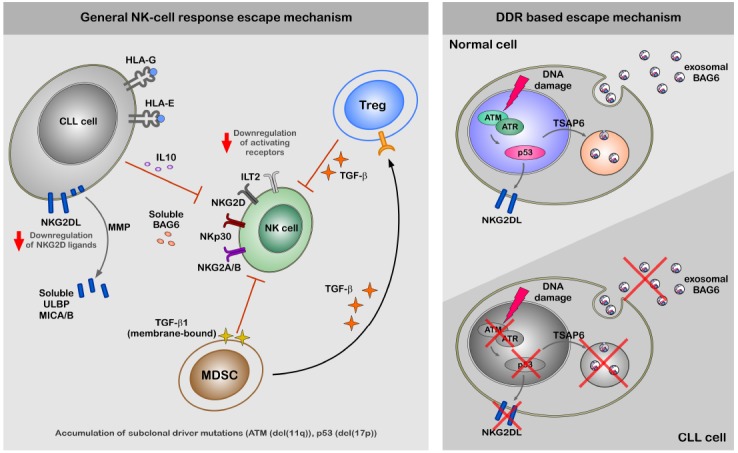
**Figure 1. A hypothetical model of CLL escape from NK response.** The left panel of the figure demonstrate general mechanism of the escape from NK-cell response by CLL cells. The escape of CLL cells from the NK cell response is regulated at different levels; (1) diminished expression NKG2D ligands on the cell surface, (2) increased levels of ligands for inhibitory receptors ILT2 and NKG2A/B, (3) shedding by MMPs and production of soluble ligands for activating receptors (ULBP2, MICA, and BAG6) NKG2D and NKp30 (Iclozan et al., 2013), which rather suppress than activate NK cells. Indirect suppression of NK cell activity might be regulated by MDSC and Treg cells via production of TGFβ and IL-10, which modulate expression levels of activating receptors on the cell surface of NK cells. The right panel of the figure is focused on the impaired DDR and its role for the escape from NK-cell response. Induction of DDR in healthy cells results in activation ATM-p53 axis. Activation of p53 results in cell surface expression of NKG2D ligands and exosomal release of BAG6 following transcriptional activation of TSAP6. Cell surface expression of NKG2D ligands and exosomal expression of BAG6 is impaired in CLL cells due to defects in DDR.

The expression of NKG2D ligands in response to genotoxic stress and stalled DNA replication forks is induced through canonical DDR in an ATM/ATR-dependent fashion in mouse and human fibroblasts (Gasser et al., 2005). The NKp30 ligand BAG6 is released by stressed cells via the exosomal pathway and has to be associated with these small membrane vesicles to properly activate NK cells (Simhadri et al., 2008). The release of exosomes is known to be regulated by TSAP6 in a p53-dependent manner (Lespagnol et al., 2008). Thus, defects in the DDR such as p53 mutations may directly affect NK cell-dependent recognition and elimination of CLL cells (Reiners et al., 2013). In line, an impaired expression of ligands for two major activating receptors—NKG2D and NKp30—was shown to be associated with CLL probably explaining NK cell anergy in this disease (Figure 1; Salih et al., 2008; Nuckel et al., 2010; Costello et al., 2012; Reiners et al., 2013). However, mechanisms of escape from NK response in CLL are not completely clear, but defects in NK cell activity strongly correlate with progression of the disease (Ziegler et al., 1981; Riches and Gribben, 2013).

## IMMUNE MICROENVIRONMENT DRIVES CLL PROGRESSION

The tumor microenvironment plays an important role in CLL progression. The functional components of the microenvironment can be divided in three groups. The first group includes bone marrow stromal cells (BMSC), nurse-like cells (NLC), and follicular dendritic cells (FDCs), collectively involved in supporting selection, survival and proliferation of CLL cells. BMSCs send anti-apoptotic signals via VCAM and integrins and protect CLL cells against conventional chemotherapy (Gehrke et al., 2011). NLC attract CLL cells by secreting CXCL12 and CXCL13 and protect from drug induced apoptosis via CXCL12, BAFF, APRIL, CD31, and plexin-B via activation of prosurvival cascades such as NFκB and ERK (Burger and Gribben, 2014). Also NLCs play important role in activation of the BCR signaling cascade. FDCs protect CLL cells from apoptosis by direct contact resulting in upregulation of antiapoptotic protein MCL-1 (Endo et al., 2007).

The second group is represented by regulatory T cells (Treg) and myeloid-derived suppressor cells (MDSCs). They interfere with the complex interaction between the immune system and transformed cells via production of immunosuppressive soluble factors such as TGF-β and IL-10 (D’Arena et al., 2013; Jitschin et al., 2014).

The third group encompasses components of the immune system, including CD4^+^ T, CD8^+^ T, and NK cells. Despite an elevated T cell count in the peripheral blood, the T cell compartment is abnormal in CLL, showing profound functional defects and signs of chronic activation [upregulation of CD69, HLA-DR, and CD57 and downregulation of CD28 and CD62L (Pedersen et al., 2002; Burger and Gribben, 2014)]. CD4^+^ T cells stimulate CLL cells via CD40/CD40L crosstalk to induce the production of CCL17 and CCL22 for attracting Th2 lymphocytes (Nakayama et al., 2004). The NK cell subset in CLL patients shows reduced ability to attack cancer cells partly owing to diminished expression of the activating NK receptor NKp30 on the cell surface (Costello et al., 2012). Also, HLA-G and HLA-E on the CLL cells and high levels of soluble/decoy ligands, for activating NK cell receptors suppress NK cell function (Nuckel et al., 2005).

## NOVEL APPROACHES FOR CLL TREATMENT: IMMUNE CONSTRUCTS AS TOOLS TO REDIRECT NK CELLS AGAINST TUMOR CELLS

The improved understanding of the pathology of CLL and the role of the microenvironment resulted in the development of novel less toxic agents (Burger and Gribben, 2014; Yair et al., 2014). These new compounds include inhibitors aimed at BCR signaling pathway (Pallasch and Hallek, 2014), antiapoptotic proteins (Yair et al., 2014), mAbs (Robak, 2013), and immune-modulatory drugs (Burger and Gribben, 2014; Yair et al., 2014).

Current immunotherapy for CLL is mainly intended to stimulate T cells in order to eliminate the tumor (Costello et al., 2012; Burger and Gribben, 2014), whereas NK cell-based therapies are not as advanced. Various recombinant immunoligands and immune constructs to restore impaired NK cell activity have been developed and analyzed pre-clinically. While both represent recombinant constructs, the immunoligands utilize natural ligands for immune receptors fused to tumor-specific single chain variable fragment (scFv) or other antibody-derived fragments whereas the immune constructs utilize antibody-derived components to target both immune and target cells (Vyas et al., 2014). So far, the activating receptors FcγRIIIa, NKG2D, and NKp30 were used as target structures on NK cells.

FcγRIIIa (CD16a) s is one of the main NK cell-activating receptors, which upon stimulation, mediates ADCC through the release of granzyme and perforin (Alderson and Sondel, 2011). The clinical success of many FDA-approved mAbs (e.g., Rituximab) is partially attributed to NK cell-mediated ADCC through FcγRIIIa receptor (Houot et al., 2011). Several bispecific and trispecific immunoconstructs with one arm specific for the human FcγRIIIa receptor have been developed. These constructs target different tumor antigens (CD33, CD123, and CD19) and are currently tested for effectiveness (Singer et al., 2010; Stein et al., 2010).

NKG2D is known as the most important receptor involved in immune evasion of CLL and other tumor cells (Huergo-Zapico et al., 2014). Tumor cells downregulate the expression of the ligands for NKG2D and release soluble ligands upon induced shedding by matrix metalloproteinases (MMPs) that block NKG2D receptor function (Huang et al., 2011; Chitadze et al., 2013). Immunoligands containing human NKG2D ligands have already been generated (Germain et al., 2005; Pogge von Strandmann et al., 2006; Jachimowicz et al., 2011; Kellner et al., 2012b; Rothe et al., 2014). One of them is a recombinant bispecific immunoligand bearing ULBP2 ligand–scFv fusion in a single chain format. The first construct of this kind was ULBP2-BB4, which links NK cells and CD138^+^ tumor cells through the ULBP2 ligand and the BB4 scFv with specificity for the TAA CD138 (Pogge von Strandmann et al., 2006). ULBP2-BB4 successfully activated and retargeted NK cells against multiple myeloma (MM) tumor cell lines and primary patient tumor cells and showed antitumor activity in a xenograft MM model. Another bispecific protein developed consists of recombinant MICA as NKG2D ligand chemically conjugated to Fab fragments from mAb specific for TAAs such as CD19 and CD20 (Kellner et al., 2012b). Malignant cells, which are otherwise resistant, can be rendered susceptible to NK cell attack by NKG2D ligand MICA in this format (Germain et al., 2005).

**FIGURE 2:**
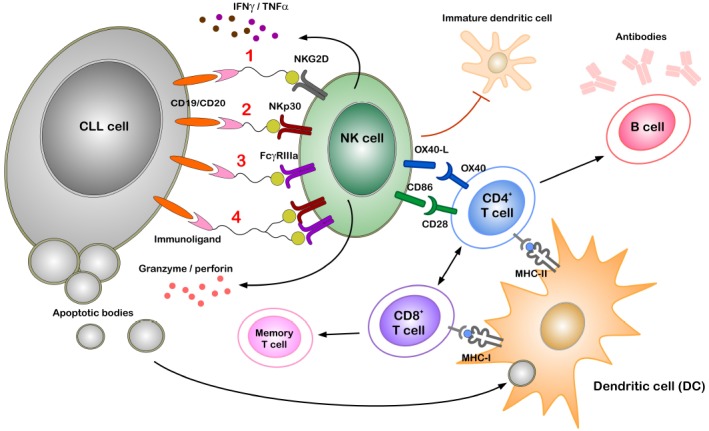
**Figure 2. Potential role of immunoligands to redirect NK cells to CLL cells.** Immunoligands with specificities for CLL cells (through CD19 or CD20) and for NK cells [through activating receptors on NK cells such as NKG2D, NKp30, FcγRIIIa or simultaneous NKp30 and FcγRIIIa(1–4)] can link and activate respective immune cells even in the presence of active immune suppression. Stimulating of either of the activating receptors on NK cell leads to cytokine secretion (IFNγ, TNF*α*) and degranulation, thereby killing tumor cells by apoptosis. Professional antigen presenting cells (APCs) such as dendritic cells (DCs) phagocytose components of dying tumor cells and present tumor antigens to both CD8^+^ and CD4^+^ T cells, thereby inducing cell-mediated and humoral adaptive immunity and memory response. Direct NK–DC crosstalk in terms of maturation of DCs and killing of immature DCs is mainly attributed to NKG2D and NKp30 activation. Additionally, following activation NK cells express OX40 ligand (OX40L) and CD86 on the cell surface, which can bind to the co-stimulatory receptors OX40 and CD28 expressed by CD4^+^ T cells. Direct interaction between NK and CD4^+^ T cells through such co-stimulatory molecules can enhance T cell effector functions.

Targeting NKp30 to reactivate NK cells in CLL is most promising and feasible as novel activating ligands for NKp30 are identified recently. While BAG6 can activate NK cells via NKp30 to induce target killing of tumor cells and immature DCs when expressed on the surface of exosomes (Pogge von Strandmann et al., 2007; Simhadri et al., 2008), expression of B7-H6 seems to be more tumor-confined as it is also found on the surface of cancer cells (Brandt et al., 2009; Matta et al., 2013). A bispecific immunoligand (B7-H6-7D8) comprising the B7-H6 (NKp30 ligand) ectodomain fused to a 7D8-derived anti-CD20 scFv was generated (Kellner et al., 2012a). The B7-H6-7D8 immunoligand efficiently redirected NKp30-dependent NK cell mediated lysis toward CD20^+^ lymphoma cells (Kellner et al., 2012a, 2013). Furthermore, this construct enhanced the lysis of target cells when used along with either rituximab (anti-CD20 mAb which activates the FcgRIIIa receptor) or NKG2D activating construct ULBP2-7D8 (Kellner et al., 2012a, 2013). The concept of synergistic activation of NK cells by multiple activating receptors is proven in various systems *in vitro* (Bryceson et al., 2006; Morgado et al., 2011; Deguine et al., 2012) and will be applied in the next generation of immunoligands in a triplebody format (Vyas et al., 2014; Figure 2).

## CONCLUDING REMARKS AND FUTURE PERSPECTIVES

In the last decade a better understanding of the role of the immune environment in progression of CLL led to new targeted therapy options. NK cells are the first line to attack the cancer, but their activity is highly impaired in CLL patients. Recent studies show impressive pre-clinical responses to immunoligands and immunoconstructs redirecting NK cells against different cancers. Novel immune construct-mediated restoration of NK cell functionality in CLL patients is an attractive strategy for treatment, which might even pave the way for recovery of patients with high risk cytogenetic aberrations and/or refractory disease.

### Conflict of Interest Statement

The authors declare that the research was conducted in the absence of any commercial or financial relationships that could be construed as a potential conflict of interest.
